# Sickness absence > 14 days following mild traumatic brain injuries from traffic accidents: a Swedish nationwide register study

**DOI:** 10.1186/s12889-025-22704-5

**Published:** 2025-04-24

**Authors:** Christian Oldenburg, Linnea Kjeldgård, Helena Stigson, Emilie Friberg

**Affiliations:** 1 Division of Insurance Medicine, Department of Clinical Neuroscience, Karolinska Institutet, Stockholm, SE–171 77 Sweden; 2 Folksam Insurance Group, Stockholm, Sweden

**Keywords:** Concussion, Sick leave, Population-based, Pedestrians

## Abstract

**Background:**

Mild traumatic brain injuries (mTBI), including concussions, following traffic accidents is common. How often these injuries lead to sickness absence (SA) among working aged individuals is however insufficiently studied. Thus, the aim of this study was to examine frequency of new SA following mTBI sustained in a road traffic environment and its associations with sociodemographic and injury-related factors.

**Method:**

Nationwide population-based register study. Working aged individuals (18–63), living in Sweden, who in 2014 to 2016 acquired an mTBI in a traffic accident were included based on in- and specialised out-patient health care records. Information on SA (> 14 days), disability pension, pre-injury factors (age, sex, education, marital status, type of living area, country of birth, income from work) as well as injury-related factors (type of road user, in- or outpatient health care) were used in analyses of risk factors for a new SA-spell. Odds ratios (ORs), both crude and adjusted, with 95% confidence intervals (CIs) were estimated with logistic regression.

**Results:**

6073 individuals were identified. 12% had a new SA spell after injury. Sociodemographic risk factors were female sex, older age and being born outside Sweden. Car occupants had higher ORs for new SA, compared to pedestrians, bicyclists, and other road users, and was also associated with longer duration spells (> 90 days). Having received in-patient health care was associated with an OR of 3.7 for new SA compared to those only receiving out-patient health care (including emergency department visits). Having received in-patient health care was also associated with longer duration spells.

**Conclusion:**

A traffic related mTBI is most often a benign injury, seldom resulting in a new SA spell of longer duration. When it does, it is more likely to involve car occupants, and those who have required in-patient health care.

## Background

Traumatic brain injury (TBI) is an “acute brain injury resulting from mechanical energy to the head from external physical forces” [1]. It remains a significant global health concern with an estimated worldwide incidence of 69 million new annual cases [2]. The vast majority of all TBI: s are considered mild (mTBI), amounting to over 90% of all cases [3]. Classification of severity is based on the patients status upon presenting to health care, most commonly by use of the Glasgow Coma Scale (GCS) [4]. Not all mTBI: s are associated with visible signs of intracranial injury on day of injury computerised scans or magnetic resonance imaging, thus a common distinction is often made between complicated and uncomplicated mild TBI [5, 6, 7], the latter synonymous with concussion. The sheer volume of mTBI is frequently referred to as a “silent epidemic” [8, 9, 10], because sequelae is predominantly cognitive and emotional in nature and not always immediately apparent for the surrounding people. Common symptoms after mTBI includes headache, attention problems, irritability and anxiety which usually resolves within days or weeks after injury [11]. However, a substantial minority will continue to report unresolved symptoms for months and even years [12, 13, 14, 15].

One major cause for mTBI is road traffic accidents [16, 17, 18]. Road traffic accidents causing physical injury (of any kind) may lead to a wide range of psychosocial consequences and cause significant distress [19, 20]. In particular, returning to work (RTW) if the injury has caused a temporary sick leave may be the most pressing activity to resume. Very few studies have examined RTW rates for individuals who sustained an mTBI in a road traffic accident specifically. Friedland and Dawson [21] found that by six months after injury only 42% of their mTBI subjects (*n* = 64) had returned to work. A recent retrospective TBI-study by Thor et al. [22] found that 64% of mTBI patients had returned to work by the time the case files were examined, on average two years after injury. There are two systematic reviews on RTW after mTBI in general that shows better rates. In 2014, the International Collaboration on Mild Traumatic Brain Injury Prognosis (iCOMP) included four studies, deemed scientifically admissible, that reported RTW or employment outcome after mTBI. This review concluded that most workers return to work within three to six months after injury but that a minority of 5 to 20% face persisting problems 1–2 years after injury [23]. In 2018 Bloom et al. included 14 studies in their review and reported an RTW-rate of 50% by one month and 80% by six months after injury [24].

Studies addressing RTW on mTBI patient are faced with numerous challenges, and sometimes fall short. In both the above mentioned systematic reviews [23, 24] several issues are highlighted, such as often unclear definitions of RTW, small sample sizes, only single-site studies, varying compensation systems, use of self-report data, selection bias and lack of control of pre-injury status. Some of these issues could be remediated by use of nationwide registers with data from health care and sickness absence (SA) spells following injury. This will allow for studying groups of mTBI patients of adequate size, selected through registers rather than self-selected samples. Using unbiased registry data will also allow for control of pre-injury status and detailed outcome with regards to SA spells with exact extension of reimbursed days. RTW and SA is not the same. In many compensation systems (including the Swedish), SA can be granted also to people who are unemployed or studying. Registry data on SA thus have the potential to capture a more complete view of the consequences of mTBI.

The aim of the present study was to examine SA for working aged individuals with mTBI caused by a road traffic accident. To get the full extent of road traffic accidents we also included accidents to pedestrians in road environments [25]. Furthermore, we wanted to explore risk factors for SA, both generally and for different lengths of SA by different sociodemographic and injury-related factors.

## Method

### Study design

We conducted a population-based register study of SA among working aged individuals (18–63), living in Sweden during the years 2014–2016 who sustained a new mTBI in a road traffic accident.

### The Swedish context

Swedish laws and regulations regarding SA states that all residents, ≥ 16 years old, who have income from work, unemployment or parental leave can apply for SA benefits from the public Swedish Social Insurance Agency if they have a disease or an injury that has caused a reduction of their work capacity. The first day of a SA-spell is a qualifying day and not reimbursed, self-employed can opt for more qualifying days. If the SA-spell lasts longer than seven days, a medical certificate from a physician is required. For most employee’s reimbursement for SA is provided by the employer for the first 14 days. However, for others (e.g., unemployed), the Social Insurance Agency will provide reimbursements from day 2 of the SA-spell. This means that data on shorter SA spells (i.e., < 14 days) is available for these individuals from the agency’s register. In order to not introduce bias, we chose to only include information on SA spells lasting longer than 14 days. SA can be granted for full time or part time (100, 75, 50, 25%) of ordinary working hours. Disability pension (DP) can be granted individuals in ages 19–64 if their injury or disease have resulted in long term or permanent reduction of their work capacity. DP can also be granted full time, or in part-time increments just as SA. This means that an individual on part-time DP can have a simultaneous SA on part time. Calculation of mean and median net days of SA was summed to whole days (e.g., SA for 50% for two gross days would count as 1 net day).

### Data sources

Micro-data from five nationwide registers, from three authorities was used. The Longitudinal integration database for health insurance and labour market studies (LISA) from Statistics Sweden was used to identify all individuals living in Sweden in the years 2014 to 2016. LISA was also used to obtain sociodemographic information (sex, age, educational level, country of birth, type of living area, marital status, and having had income from work). The national patient register, from the Swedish National Board of Health and Welfare, holds information on all inpatient health care as well as all specialised out-patient health care, including visits to emergency departments. Main diagnosis, eventual secondary diagnoses and external cause of morbidity is recorded according to the International Statistical Classification of Diseases and related Health Problems; ICD-10 [26]. The external validity of registered concussion diagnosis in the Swedish in-patient register is excellent with 100% positive predictive value, and 99,8% negative predictive value [27], however, no external validation of registered concussion diagnoses in specialized out-patient settings have been performed. The Cause of Death Register from the same authority was used to identify those who had died within the first 30 days after injury. Finally, The Micro Data for Analysis of the Social Insurance (MiDAS) from the Swedish Social Insurance Agency was used for information on dates, extent, and length of SA and DP. All data were linked at the individual level, using the unique personal identity number assigned to all residents in Sweden [28].

### Study population

We began by identifying all patients within the patient registers who had been diagnosed with concussion (S06.0) due to an accident while in transportation (external cause of morbidity code V01-99). Road users were then categorized as pedestrians (code V01-09), bicyclists (code V10-19), or car occupants (code V40-49). To ensure the inclusion of pedestrians injured in fall accidents on roads, we expanded our scope to include accidents that occurred on “street and highway” (W00.4, W01.4, W02.4, W03.4, W04.4, W05.4, W10.4, W15.4, W17.4, W18.4, W19.4), and “striking against or bumped into by another person, street and highway” (W51.4). Finally, all other morbidity codes formed a final category of other road users (e.g., motorcycle riders, bus occupants).

We considered the date of the in- or specialized out-patient healthcare visit as the date of injury, as the actual date of injury is not available in the register data. In total, we identified 7,154 working-age individuals who had received health care for concussion caused by a road traffic accident during 2014–2016 when aged 18 to 63. To be included the individual had to live in Sweden 31 December the year before the injury. To include only new injuries, we excluded patients who had received any in- or specialized outpatient health care for any traumatic brain injury (S06) in the 365 days prior to the injury date (*n* = 187). We also excluded those who had concussion only as a secondary diagnosis (*n* = 894).

Sociodemographic variables were measured using register data from the year preceding the injury, and were categorized by sex (women, men), age (18–24, 25–34, 45 − 44, 45–54, 55–63 years), educational level (elementary school including missing, high school 10–12 years, university or college > 12 years), country of birth (Sweden, not Sweden), type of living area (cities, towns and suburbs, and rural areas) based on Eurostat’s classification for degree of urbanization [29], marital status (married, not married), having received income from work (yes, no). The reference groups for the regression analysis were selected based on having the lowest expected proportions of new SA (e.g., male sex) or, in some cases, being the largest group (e.g., individuals born in Sweden). While the proportion of new SA was anticipated to be lowest in the youngest age group (18–24 years), we chose the 25–34 age group as the reference because many individuals in the youngest group are likely to be students and may not even be eligible for SA benefits.

### Statistical analysis

Basic demographics (sex, age, education level, country of birth, type of living area, marital status), type of injury and inpatient vs. outpatient received health care were shown by SA and DP status at the time of injury, using descriptive statistics. Odds ratios (OR) with 95% confidence intervals (CI) for starting a new SA in connection with the injury were calculated by binary logistic regression. For these analyses, individuals with an already ongoing SA or full-time DP were excluded, because they were not at risk for a new SA spell. Multinomial logistic regression was used to analyse the association between sociodemographic and injury related factors with different durations of SA (< 30 net days, 30–90 net days, > 90 net days). Crude and mutually adjusted ORs were calculated. The statistical analyses were performed using SPSS (version 28) and SAS (version 9.4). The project was approved by the Regional Ethical Review Board in Stockholm, Sweden.

## Results

A total of 6073 individuals between the ages of 18–63 received in- or specialized outpatient healthcare for mTBI sustained in a traffic accident during the years 2014 to 2016. The sex distribution was approximately equal, with a slightly higher proportion of women than men. The mean age of the study population was 35.6 (SD 13.4), and the distribution skewed towards the younger age groups, as shown in Table 1. Most individuals were born in Sweden, unmarried, had at least a high school education, lived in urban areas, and were employed.

**Table 1:** Sociodemographic and injury characteristics for individuals. Aged 18–63 with mild traumatic brain injury sustained in a traffic accident. By sickness absence (SA) and disability pension (DP) status at the time of injury

	No New SA		New SA (> 14 days)		Ongoing SA		Ongoing full-time DP		Total	
	*N*	%	*N*	%	*N*	%	*N*	%	*N*	%
**Total**	4736	78.0	737	12.1	362	6.0	238	3.9	6073	100.0
**Sex**										
Women	2402	75.9	409	12.9	230	7.3	124	3.9	3165	52.1
Men	2334	80.3	328	11.3	132	4.5	114	3.9	2908	47.9
**Age group**,** years**										
18–24	1639	90.5	100	5.5	28	1.5	44	2.4	1811	29.8
25–34	1043	77.9	175	13.1	89	6.6	32	2.4	1339	22.0
35–44	843	72.9	183	15.8	113	9.8	17	1.5	1156	19.0
45–54	739	70.0	173	16.4	86	8.2	57	5.4	1055	17.4
55–63	472	66.3	106	14.9	46	6.5	88	12.4	712	11.7
**Education level**										
Elementary school	925	78.5	97	8.2	52	4.4	105	8.9	1179	19.4
High school	2329	77.7	368	12.3	194	6.5	108	3.6	2999	49.4
University/College	1482	78.2	272	14.4	116	6.1	25	1.3	1895	31.2
**Born in Sweden**										
No	688	73.3	133	14.2	65	6.9	52	5.5	938	15.4
Yes	4048	78.8	604	11.8	297	5.8	186	3.6	5135	84.6
**Marital status**										
Not married	3589	80.0	465	10.4	233	5.2	201	4.5	4488	73.9
Married	1147	72.4	272	17.2	129	8.1	37	2.3	1585	26.1
**Type of living area**										
Cities	1802	78.7	269	11.7	137	6.0	83	3.6	2291	37.7
Towns and suburbs	2018	77.8	322	12.4	157	6.1	97	3.7	2594	42.7
Rural areas	916	77.1	146	12.3	68	5.7	58	4.9	1188	19.6
**Income from work**										
No	1261	78.7	49	3.1	66	4.1	227	14.2	1603	26.4
Yes	3475	77.7	688	15.4	296	6.6	11	0.2	4470	73.6
**Type of road user**										
Pedestrians	618	73.7	78	9.3	65	7.7	78	9.3	839	13.8
Bicyclists	1634	81.3	225	11.2	87	4.3	65	3.2	2011	33.1
Car occupants	952	73.6	202	15.6	90	7.0	49	3.8	1293	21.3
Other road users	1532	79.4	232	12.0	120	6.2	46	2.4	1930	31.8
**Inpatient health care**										
No	3402	81.0	377	9.0	259	6.2	164	3.9	4202	69.2
Yes	1334	71.3	360	19.2	103	5.5	74	4.0	1871	30.8

Most individuals (78%) did not have a new SA (> 14 days) after their injury and were not on SA or full-time DP at the time of the injury. A total of 12% initiated a new SA spell that lasted more than 14 days, while 10% were already on SA or full-time DP at the time of the injury. The proportion of mTBI was highest among bicyclists, accounting for 33% of cases. In-patient healthcare was received by nearly one-third of the study population.

A total of 5473 individuals who were at risk of a new SA (i.e., excluding individuals with full-time DP or ongoing SA at the time of injury) were included in the analysis of odds ratios (ORs), as shown in Table 2. After adjusting for all factors, several sociodemographic factors were found to be associated with new SA. Women were found to be at higher risk of receiving a new SA compared to men. The older age groups were also found to have a higher risk for new SA, although only the OR for the 45–54 age group was significantly higher than the reference group. Individuals born abroad had an elevated OR for new SA. The youngest age group (18–24) and individuals without income from work had significantly lower odds.

**Table 2:** Odds ratios (OR) and confidence intervals (CI) for new sickness absence (SA) following a mild traumatic brain injury sustained in a traffic accident, by sociodemographic and injury characteristics among the 5473 injured individuals without already ongoing SA or full-time disability pension

	*n*	% new SA (> 14 days)	Crude OR (95% CI)	Adjusted* OR (95% CI)
**Sex**				
Women	2811	14.5	1.21 (1.04–1.42)	1.44 (1.22–1.71)
Men	2662	12.3	ref.	ref.
**Age group**,** years**				
18–24	1739	5.8	0.36 (0.28–0.47)	0.44 (0.34–0.58)
25–34	1218	14.4	ref.	ref.
35–44	1026	17.8	1.29 (1.03–1.62)	1.25 (0.98–1.59)
45–54	912	19.0	1.40 (1.11–1.76)	1.30 (1.02–1.67)
55–63	578	18.3	1.34 (1.03–1.74)	1.29 (0.97–1.72)
**Education level**				
Elementary school	1022	9.5	0.57 (0.45–0.73)	1.12 (0.85–1.49)
High school	2697	13.6	0.86 (0.73–1.02)	1.10 (0.91–1.32)
University/College	1754	15.5	ref.	ref.
**Born in Sweden**				
No	821	16.2	1.30 (1.06–1.59)	1.32 (1.05–1.66)
Yes	4652	13.0	ref.	ref.
**Marital status**				
Not married	4054	11.5	0.55 (0.46–0.64)	0.86 (0.71–1.04)
Married	1419	19.2	ref.	ref.
**Type of living area**				
Cities	2071	13.0		
Towns and suburbs	2340	13.8	1.07 (0.90–1.27)	1.02 (0.84–1.22)
Rural areas	1062	13.7	1.07 (0.86–1.33)	0.96 (0.76–1.22)
**Income from work**				
No	1310	3.7	0.20 (0.15–0.26)	0.23 (0.17–0.31)
Yes	4163	16.5	ref.	ref.
**Type of road user**				
Pedestrians	696	11.2	ref.	ref.
Bicyclists	1859	12.1	1.09 (0.83–1.43)	0.93 (0.70–1.24)
Car occupants	1154	17.5	1.68 (1.27–2.26)	1.79 (1.33–2.41)
Other road users	1764	13.2	1.20 (0.91–1.58)	1.18 (0.88–1.57)
**Inpatient health care**				
No	3779	10.0	ref.	ref.
Yes	1694	21.3	2.44 (2.08–2.85)	2.62 (2.21–3.09)

*Adjusted for all factors

Regarding accident and injury characteristics, individuals who received in-patient health care were more likely to initiate a new SA compared to those who only received specialized out-patient health care. Similarly, car occupants had higher odds of new SA than pedestrians, whereas no significant differences were observed for bicyclists or other road users.

Using multinomial logistic regression, crude and adjusted ORs were calculated for different durations of SA (Table 3). Women had a higher risk of new SA than men for short (< 30 days) and medium (30–90 days) duration SA spells, while there were no significant differences for long duration (> 90 days) spells. All older age groups also had a higher risk of long duration SA compared to the reference age group. Regarding injury-related factors, those who had received in-patient healthcare had a significantly higher risk of short, medium, and long-term SA than those who had only received outpatient health care. Having been injured as a car occupant was associated with a higher risk of SA for medium and long-term duration spells compared to pedestrians.

**Table 3:** Multinomial logistic regression showing odds ratios (OR) and confidence intervals (CI) for different duration of new SA for individuals with mild traumatic brain injury sustained in a traffic accident, by sociodemographic and injury characteristics

	No New SA		SA 15–29 Net Days				SA 30–90 Net Days				SA > 90 Net Days			
	n	%	n	%	Crude	Adjusted*	n	%	Crude	Adjusted*	n	%	Crude	Adjusted*
**Total**	4736	86.5	257	4.7			304	5.6			176	3.2		
**Sex**														
Women	2402	50.7	160	62.3	1.60 (1.24–2.08)	1.81 (1.38–2.38)	161	53.0	1.09 (0.87–1.38)	1.30 (1.02–1.66)	88	50.0	0.97 (0.72–1.31)	1.26 (0.91–1.73)
Men	2334	49.3	97	37.7	ref.	ref.	143	47.0	ref.	ref.	88	50.0	ref.	ref.
**Age group**,** years**														
18–24	1639	34.6	27	10.5	0.26 (0.19–0.42)	0.35 (0.22–0.56)	48	15.8	0.39 (0.27–0.57)	0.45 (0.30–0.65)	25	14.2	0.50 (0.29–0.84)	0.63 (0.36–1.09)
25–34	1043	22.0	65	25.3	ref.	ref.	78	25.7	ref.	ref.	32	18.2	ref.	ref.
35–44	843	17.8	62	24.1	1.18 (0.82–1.69)	1.03 (0.70–1.50)	76	25.0	1.21 (0.87–1.68)	1.25 (0.89–1.77)	45	26.6	1.74 (1.10–2.76)	1.79 (1.10–2.92)
45–54	739	15.6	60	23.3	1.30 (0.91–1.87)	1.09 (0.74–1.61)	67	22.0	1.21 (0.86–1.70)	1.26 (0.88–1.80)	46	26.1	2.03 (1.28–3.22)	1.97 (1.20–3.21)
55–63	472	10.0	43	16.7	1.46 (0.98–2.18)	1.25 (0.81–1.92)	35	11.5	0.99 (0.66–1.50)	1.08 (0.70–1.67)	28	15.9	1.93 (1.15–3.25)	2.03 (1.16–3.55)
**Education level**														
Elementary school	925	19.5	25	9.7	0.37 (0.24–0.58)	0.88 (0.54–1.42)	43	14.1	0.63 (0.44–0.91)	1.10 (0.73–1.64)	29	16.5	0.83 (0.53–1.31)	1.63 (0.99–2.71)
High school	2329	49.2	125	48.6	0.74 (0.57–0.97)	1.06 (0.79–1.41)	152	50.0	0.89 (0.69–1.14)	1.07 (0.81–1.41)	91	51.7	1.03 (0.74–1.45)	1.28 (0.88–1.84)
University/College	1482	31.3	107	41.6	ref.	ref.	109	35.9	ref.	ref.	56	31.8	ref.	ref.
**Born in Sweden**														
No	688	14.5	44	17.1	1.22 (0.87–1.70)	1.19 (0.83–1.71)	55	18.1	1.30 (0.96–1.76)	1.37 (0.99–1.91)	34	19.3	1.41 (0.96–2.07)	1.41 (0.93–2.16)
Yes	4048	85.5	213	82.9	ref.	ref.	249	81.9	ref.	ref.	142	80.7	ref.	ref.
**Marital status**														
Not married	3589	75.8	149	58.0	0.44 (0.34–0.57)	0.71 (0.53–0.95)	210	69.1	0.71 (0.56–0.92)	1.09 (0.82–1.44)	106	60.2	0.48 (0.36–0.66)	0.79 (0.55–1.11)
Married	1147	24.2	108	42.0	ref.	ref.	94	30.9	ref.	ref.	70	39.8	ref.	ref.
**Urbanization**														
Cities	1802	38.0	96	37.4	ref.	ref.	110	36.2	ref.	ref.	63	35.8	ref.	ref.
Towns and suburbs	2018	42.6	124	48.2	1.15 (0.88–1.52)	1.14 (0.85–1.51)	121	39.8	0.98 (0.75–1.28)	0.96 (0.73–1.26)	77	43.8	1.09 (0.78–1.53)	0.94 (0.66–1.35)
Rural areas	916	19.3	37	14.4	0.76 (0.52–1.12)	0.73 (0.49–1.10)	73	24.0	1.31 (0.96–1.77)	1.22 (0.88–1.69)	36	20.5	1.12 (0.74–1.71)	0.86 (0.55–1.34)
**Income from work**														
No	3475	73.4	244	94.9	0.15 (0.08–0.26)	0.20 (0.11–0.35)	274	90.1	0.30 (0.21–0.44)	0.34 (0.23–0.51)	170	96.6	0.10 (0.04–0.22)	0.10 (0.04–0.23)
Yes	1261	26.6	13	5.1	ref.	ref.	30	9.9	ref.	ref.	6	3.4	ref.	ref.
**Type of road user**														
Pedestrians	618	13.0	34	13.2	ref.	ref.	31	10.2	ref.	ref.	13	7.4	ref.	ref.
Bicyclists	1634	34.5	86	33.5	0.96 (0.64–1.44)	0.81 (0.53–1.24)	95	31.3	1.16 (0.77–1.76)	1.01 (0.66–1.55)	44	25.0	1.28 (0.68–2.39)	1.06 (0.56–2.01)
Car occupants	952	20.1	61	23.7	1.17 (0.76–1.79)	1.38 (0.88–2.17)	79	26.0	1.65 (1.08–2.54)	1.65 (1.06–2.58)	62	35.2	3.10 (1.69–5.68)	3.28 (1.75–6.14)
Other road users	1532	32.3	76	29.6	0.90 (0.60–1.37)	0.89 (0.58–1.37)	99	32.6	1.29 (0.85–1.95)	1.24 (0.81–1.90)	57	32.4	1.77 (0.96–3.25)	1.80 (0.96–3.37)
**Inpatient health care**														
No	3402	71.8	145	56.4	ref.	ref.	159	52.3	ref.	ref.	73	41.5	ref.	ref.
Yes	1334	28.2	112	43.6	1.97 (1.53–2.54)	2.23 (1.71–2.91)	145	47.7	2.33 (1.84–2.94)	2.46 (1.93–3.13)	103	58.5	3.60 (2.65–4.89)	3.71 (2.70–5.11)

*adjusted for all factors

## Discussion

This nationwide register study investigated the incidence of new SA spells lasting longer than 14 days among 6073 working-age adults who received medical care for a mTBI resulting from a traffic accident. Of those, 12% had a new SA spell (> 14 days), while 10% were already on SA or full-time DP at the time of injury. Our analysis found that females were more likely to have a new SA spell, but there were no differences in the risk of long-duration SA (> 90 days) between sexes. Older age (> 34 years of age) was associated with new SA in particular with long duration SA. *Increasing age was also associated with a higher frequency of ongoing SA and DP at the time of injury*,* which aligns with general workforce trends* [30]. *However*,* the prevalence observed in our study was lower than expected based on official workforce data*,* suggesting a reduced risk of RTI in this group. This may reflect differences in traffic exposure*,* as individuals on SA/DP likely travel less frequently than those actively working.* We also found a higher risk for new SA among individuals born outside of Sweden.

Regarding injury-related factors, our data was limited to healthcare setting (in- vs. outpatient) and type of road user (pedestrian, bicyclist, car occupant, other). However, we found that both of these factors were significantly associated with the risk of new SA. Receiving in-patient healthcare after injury more than doubled the risk of a new SA and increased the risk for long-duration SA compared to those who were treated in outpatient settings only. Additionally, being injured as a car occupant was associated with a higher risk of new SA and long-duration SA compared to injuries sustained by pedestrians.

Our study indicates that the vast majority of individuals recover relatively quickly, and by three months only a small minority of 3% is still on SA. Compared to studies on smaller clinical samples of road traffic injured mTBI patients [21, 22] this number is significantly lower. The higher number in previous studies probably reflects selection biases inherent in clinical studies. For instance, in the Friedland & Dawson study [21], the sample consisted of patients referred to concussion clinics, which obviously limits generalisation to individuals who quickly recover and declines further contact with health care.

A few register-based studies with a narrower target group have reported findings that are more consistent with our results. Ohlin et al. (2018) [31] studied bicycle crashes during a three-year period. A total of 22 045 injured individuals were included, and of those 1600 (7.3%) sustained a concussion. A new SA spell was recorded in 8.4% of these cases, only 1% had an SA spell exceeding 90 days. Kjeldgård et al. (2019) [32] studied bicycle crashes during a single year (*N* = 7643). Similarly, they reported 8.2% new SA for those who had sustained a concussion. Highest incidence of new SA (35.9%) was found for traumatic brain injuries other than concussions.

In the present study, only a small minority of 3% of adults with mTBI had a long duration SA (> 90 net days). This can be contrasted with the fact that a much larger percentage of adults with mTBI report persisting (> 3 months) symptoms. A recent systematic review and meta-analysis by Cancelliere et al. (2022) [33] found a 31% prevalence of self-reported post-concussion symptoms after three to six months after injury in adult mTBI patients. Taken together, these findings suggest that the majority of mTBI patients with persisting symptoms return to work without having fully recovered. This situation may adversely affect work capacity and contribute to stress reactions.

Indeed, Silverberg et al. (2018) [34] investigated a clinical sample of mTBI-patients and observed that a significant proportion who had resumed work by three months continued to endorse post-concussion symptoms, depression, anxiety and bodily pain and admitted to getting less work done and doing more mistakes at work. Future studies should examine if there are any long-term negative consequences of premature return to work following mTBI, considering both health-related and economic implications.

Due to limitations in the patient registers used in our study, we were unable to include mTBI patients with intracranial signs of brain injury (such as contusions or haemorrhage) due to the absence of TBI severity defining data. Therefore, our findings do not generalize to all mTBI patients. It is worth noting though that concussions constitute the largest subgroup within the broader mTBI spectrum. Among patients presenting to emergency departments with a perfect GCS score of 15, only around 5% exhibit intracranial signs of brain injury on computerized tomography (CT). This percentage increases to 20% for patients with a GCS score of 14 and further rises to 30% for those with a GCS score of 13, which represents the lower limit for mTBI [35].

Intuitively, one would assume that mTBI patients with visible signs of injury have worse outcome, but studies produce mixed results with regard to cognitive functioning [6] and self-reported symptoms [36, 37, 38]. A slower RTW has been found in some studies [39, 40], but not all [41]. A slower RTW for these patients could also reflect different sick-listing practises among doctors [42].

The main strength of this study is that it is not based on a sample of patients but instead all individuals in Sweden who matched the inclusion criteria during 2014 to 2016, virtually with no drop-outs. We used no self-reported data with its risk of possible biases, instead diagnoses were certified by physicians, and all register data came from nationwide administrative registers of acknowledged high quality [43]. However, it is important to note that the codes for external cause, which indicate the specific circumstances of the injury, may contain some level of errors. The inclusion of outpatient data may introduce bias, as the one-year washout period may not fully exclude individuals with older trauma. Future studies should aim to restrict outpatient data to ER visits only to minimize this potential bias. There are some limitations. First of all, we only utilised data on SA spells longer than 14 days. The full burden of concussion on SA is thus not captured, essentially leaving out all self-reported shorter spells that were not accompanied by a mandatory doctor’s certificate. Second, register data are limited to in- and specialised outpatient health care and do not capture visits to general practitioners, which narrowed our possibility to include all patients with concussion. These limitations can be viewed as potential strengths of the study, restricting the inclusion to the more serious cases. Finally, the accuracy and completeness of the register data are potential sources of uncertainty, and further research is needed to evaluate the validity of outpatient data for concussion diagnoses.

## Conclusions

To conclude, using register data we found that for individuals who sustained an mTBI in a road traffic environment, SA (> 14 days) followed in 12% of the cases. Longer duration SA spells (> 90 days) were rare and found in only 3% of the cases at risk (i.e., not including individuals already at SA or disability pension at the time of injury). Car occupants and those who required hospitalisation had a higher likelihood of experiencing prolonged SA.

## Data Availability

Due to privacy regulations, the data cannot be made publicly available. In accordance with the General Data Protection Regulation, the Swedish laws SFS 2018:218, the Swedish Data Protection Act, the Swedish Ethical Review Act, and the Public Access to Information and Secrecy Act, access to this sensitive and confidential data can only be shared after a legal review, and solely with researchers who meet the established criteria for accessing this sensitive and confidential data. Readers may contact Professor Kristina Alexanderson (kristina.alexanderson@ki.se) regarding the data.

## Abbreviations

CI: Confidence interval
DP: Disability pension
GCS: Glasgow Coma Scale
mTBI: Mild traumatic brain injury
OR: Odds ratio
RTW: Return to work
SA: Sickness absence
TBI: Traumatic brain injury

## References

[1] Carroll LJ, Cassidy JD, Holm L, Kraus J, Coronado VG, Injury WHOCCTFMTB. Methodological issues and research recommendations for mild traumatic brain injury: the WHO collaborating centre task force on mild traumatic brain injury. J Rehabil Med. 2004(43 Suppl):113– 25. 10.1080/16501960410023877 15083875

[2] Dewan MC, Rattani A, Gupta S, Baticulon RE, Hung YC, Punchak M, et al. Estimating the global incidence of traumatic brain injury. J Neurosurg. 2019;130(4):1080–97. 29701556 10.3171/2017.10.JNS17352

[3] Feigin VL, Theadom A, Barker-Collo S, Starkey NJ, McPherson K, Kahan M, et al. Incidence of traumatic brain injury in new Zealand: a population-based study. Lancet Neurol. 2013;12(1):53–64. 23177532 10.1016/S1474-4422(12)70262-4

[4] Teasdale G, Jennett B. Assessment of coma and impaired consciousness. A practical scale. Lancet. 1974;2(7872):81–4. 4136544 10.1016/s0140-6736(74)91639-0

[5] Williams DH, Levin HS, Eisenberg HM. Mild head injury classification. Neurosurgery. 1990;27(3):422–8. 2234336 10.1097/00006123-199009000-00014

[6] Karr JE, Iverson GL, Williams MW, Huang SJ, Yang CC. Complicated versus uncomplicated mild traumatic brain injuries: A comparison of psychological, cognitive, and post-concussion symptom outcomes. J Clin Exp Neuropsychol. 2020;42(10):1049–58. 33161877 10.1080/13803395.2020.1841118

[7] Voormolen DC, Zeldovich M, Haagsma JA, Polinder S, Friedrich S, Maas AIR et al. Outcomes after complicated and uncomplicated mild traumatic brain injury at Three-and Six-Months Post-Injury: results from the CENTER-TBI study. J Clin Med. 2020;9(5). 10.3390/jcm9051525 PMC7291134 32443573

[8] National Center for Injury Prevention and Control CfdCaiP. Report to Congress on mild traumatic brain injury in the united States: steps to prevent a serious public health problem. Atlanta, GA; 2003.

[9] Rusnak M. Traumatic brain injury: giving voice to a silent epidemic. Nat Rev Neurol. 2013;9(4):186–7. 23478463 10.1038/nrneurol.2013.38

[10] Vaishnavi S, Rao V, Fann JR. Neuropsychiatric problems after traumatic brain injury: unraveling the silent epidemic. Psychosomatics. 2009;50(3):198–205. 19567758 10.1176/appi.psy.50.3.198

[11] Cassidy JD, Cancelliere C, Carroll LJ, Cote P, Hincapie CA, Holm LW, et al. Systematic review of self-reported prognosis in adults after mild traumatic brain injury: results of the international collaboration on mild traumatic brain injury prognosis. Arch Phys Med Rehabil. 2014;95(3 Suppl):S132–51. 24581902 10.1016/j.apmr.2013.08.299

[12] Oldenburg C, Lundin A, Edman G, Deboussard CN, Bartfai A. Emotional reserve and prolonged post-concussive symptoms and disability: a Swedish prospective 1-year mild traumatic brain injury cohort study. BMJ Open. 2018;8(7):e020884. 29982209 10.1136/bmjopen-2017-020884 PMC6042551

[13] Dikmen S, Machamer J, Temkin N. Mild traumatic brain injury: longitudinal study of cognition, functional status, and Post-Traumatic symptoms. J Neurotrauma. 2017;34(8):1524–30. 27785968 10.1089/neu.2016.4618 PMC5397200

[14] Kraus JF, Hsu P, Schafer K, Afifi AA. Sustained outcomes following mild traumatic brain injury: results of a five-emergency department longitudinal study. Brain Inj. 2014;28(10):1248–56. 24841806 10.3109/02699052.2014.916420

[15] Losoi H, Silverberg ND, Waljas M, Turunen S, Rosti-Otajarvi E, Helminen M, et al. Recovery from mild traumatic brain injury in previously healthy adults. J Neurotrauma. 2016;33(8):766–76. 26437675 10.1089/neu.2015.4070

[16] Dunne J, Quinones-Ossa GA, Still EG, Suarez MN, Gonzalez-Soto JA, Vera DS, et al. The epidemiology of traumatic brain injury due to traffic accidents in Latin America: A narrative review. J Neurosci Rural Pract. 2020;11(2):287–90. 32367985 10.1055/s-0040-1709363 PMC7195969

[17] Brazinova A, Rehorcikova V, Taylor MS, Buckova V, Majdan M, Psota M, et al. Epidemiology of traumatic brain injury in Europe: A living systematic review. J Neurotrauma. 2021;38(10):1411–40. 26537996 10.1089/neu.2015.4126 PMC8082737

[18] Walder B, Haller G, Rebetez MM, Delhumeau C, Bottequin E, Schoettker P, et al. Severe traumatic brain injury in a high-income country: an epidemiological study. J Neurotrauma. 2013;30(23):1934–42. 23822874 10.1089/neu.2013.2955

[19] Kendall E, Buys N. The psychosocial consequences of motor vehicle accidents. J Personal Interpers Loss. 1999;4(1):47–66.

[20] Craig A, Tran Y, Guest R, Gopinath B, Jagnoor J, Bryant RA, et al. Psychological impact of injuries sustained in motor vehicle crashes: systematic review and meta-analysis. BMJ Open. 2016;6(9):e011993. 27609849 10.1136/bmjopen-2016-011993 PMC5020848

[21] Friedland JF, Dawson DR. Function after motor vehicle accidents: a prospective study of mild head injury and posttraumatic stress. J Nerv Ment Dis. 2001;189(7):426–34. 11504319 10.1097/00005053-200107000-00003

[22] Thor JA, Mazlan M, Waran V. Employment status after traumatic brain injury and the effect of concomitant injuries on return to work. Brain Inj. 2021;35(8):949–56. 34096426 10.1080/02699052.2021.1934729

[23] Cancelliere C, Kristman VL, Cassidy JD, Hincapie CA, Cote P, Boyle E, et al. Systematic review of return to work after mild traumatic brain injury: results of the international collaboration on mild traumatic brain injury prognosis. Arch Phys Med Rehabil. 2014;95(3 Suppl):S201–9. 24581906 10.1016/j.apmr.2013.10.010

[24] Bloom B, Thomas S, Ahrensberg JM, Weaver R, Fowler A, Bestwick J, et al. A systematic review and meta-analysis of return to work after mild traumatic brain injury. Brain Inj. 2018;32(13–14):1623–36. 30307758 10.1080/02699052.2018.1532111

[25] Methorst R, Schepers P, Christie N, Dijst M, Risser R, Sauter D, et al. Pedestrian falls’ as necessary addition to the current definition of traffic crashes for improved public health policies. J Transp Health. 2017;6:10–2.

[26] World Health Organisation. ICD-10: international statistical classification of diseases and related health problems. Geneva, Switzerland: World Health Organisation; 1992.

[27] Ludvigsson JF, Andersson E, Ekbom A, Feychting M, Kim JL, Reuterwall C, et al. External review and validation of the Swedish National inpatient register. BMC Public Health. 2011;11:450. 21658213 10.1186/1471-2458-11-450 PMC3142234

[28] Ludvigsson JF, Otterblad-Olausson P, Pettersson BU, Ekbom A. The Swedish personal identity number: possibilities and pitfalls in healthcare and medical research. Eur J Epidemiol. 2009;24(11):659–67. 19504049 10.1007/s10654-009-9350-y PMC2773709

[29] Eurostat. Background - Degree of urbanisation - Eurostat 2023 [Available from: https://ec.europa.eu/eurostat/web/degree-of-urbanisation/background

[30] Statistics Sweden. Ohälsotalet– Sickness Absence and Disability Pension Statistics: Statistics Sweden; 2014–2016 [Available from: https://www.statistikdatabasen.scb.se

[31] Ohlin M, Kjeldgård L, Elrud R, Stigson H, Alexanderson K, Friberg E. Duration of sickness absence following a bicycle crash, by injury type and injured body region: A nationwide register-based study. J Transp Health. 2018;9:275–81.

[32] Kjeldgard L, Ohlin M, Elrud R, Stigson H, Alexanderson K, Friberg E. Bicycle crashes and sickness absence - a population-based Swedish register study of all individuals of working ages. BMC Public Health. 2019;19(1):943. 31307453 10.1186/s12889-019-7284-1 PMC6631908

[33] Cancelliere C, Verville L, Stubbs JL, Yu H, Hincapie CAA, Cassidy JD et al. Post-concussion symptoms and disability in adults with mild traumatic brain injury: a systematic review and meta-analysis. J Neurotrauma. 2022. 10.1089/neu.2022.0185 36472218

[34] Silverberg ND, Panenka WJ, Iverson GL. Work productivity loss after mild traumatic brain injury. Arch Phys Med Rehabil. 2018;99(2):250–6. 28760573 10.1016/j.apmr.2017.07.006

[35] Borg J, Holm L, Cassidy JD, Peloso PM, Carroll LJ, von Holst H et al. Diagnostic procedures in mild traumatic brain injury: results of the WHO collaborating centre task force on mild traumatic brain injury. J Rehabil Med. 2004(43 Suppl):61–75. 10.1080/16501960410023822 15083871

[36] McCauley SR, Boake C, Levin HS, Contant CF, Song JX. Postconcussional disorder following mild to moderate traumatic brain injury: anxiety, depression, and social support as risk factors and comorbidities. J Clin Exp Neuropsychol. 2001;23(6):792–808. 11910545 10.1076/jcen.23.6.792.1016

[37] Hughes DG, Jackson A, Mason DL, Berry E, Hollis S, Yates DW. Abnormalities on magnetic resonance imaging seen acutely following mild traumatic brain injury: correlation with neuropsychological tests and delayed recovery. Neuroradiology. 2004;46(7):550–8. 15185054 10.1007/s00234-004-1227-x

[38] Lannsjo M, Backheden M, Johansson U, Af Geijerstam JL, Borg J. Does head CT scan pathology predict outcome after mild traumatic brain injury? Eur J Neurol. 2013;20(1):124–9. 22812542 10.1111/j.1468-1331.2012.03813.x

[39] Huovinen A, Marinkovic I, Isokuortti H, Korvenoja A, Mäki K, Nybo T, et al. Traumatic microbleeds in mild traumatic brain injury are not associated with delayed return to work or persisting Post-Concussion symptoms. J Neurotrauma. 2021;38(17):2400–6. 33847170 10.1089/neu.2021.0055

[40] Iverson GL, Lange RT, Waljas M, Liimatainen S, Dastidar P, Hartikainen KM, et al. Outcome from complicated versus uncomplicated mild traumatic brain injury. Rehabil Res Pract. 2012;2012:415740. 22577556 10.1155/2012/415740 PMC3345249

[41] Yue JK, Phelps RR, Hemmerle DD, Upadhyayula PS, Winkler EA, Deng H et al. Predictors of six-month inability to return to work in previously employed subjects after mild traumatic brain injury: A TRACK-TBI pilot study. J Concussion. 2021;5. 10.1177/20597002211007271 PMC8153496 34046212

[42] Waljas M, Iverson GL, Lange RT, Liimatainen S, Hartikainen KM, Dastidar P, et al. Return to work following mild traumatic brain injury. J Head Trauma Rehabil. 2014;29(5):443–50. 24263178 10.1097/HTR.0000000000000002

[43] Ludvigsson JF, Almqvist C, Bonamy AK, Ljung R, Michaelsson K, Neovius M, et al. Registers of the Swedish total population and their use in medical research. Eur J Epidemiol. 2016;31(2):125–36. 26769609 10.1007/s10654-016-0117-y

